# A Case of Worsening Deep Cerebral Venous Sinus Thrombosis Managed by Intrasinus Thrombolysis

**DOI:** 10.1155/2011/272841

**Published:** 2011-10-12

**Authors:** Nidhi Sood, Nikhil Sood, Arun Talkad

**Affiliations:** ^1^Department of Internal Medicine, University of Missouri, 1 Hospital Drive, Columbia, MO 65202, USA; ^2^Department of Internal Medicine, Winthrop University Hospital, 200 Old Country Road, Mineola, NY 11501, USA; ^3^Department of Neurology, University of Illinois College of Medicine at Peoria, 530 Northeast Glen Oak, Peoria, IL 61637, USA

## Abstract

Cerebral venous sinus thrombosis (CVST) is an uncommon condition with severe consequences. Although we do not know the exact incidence and prevalence of CVST, it is an important diagnosis. Over the past decade, it has been diagnosed more frequently due to greater awareness and availability of noninvasive diagnostic techniques. Furthermore, routine diagnostic neuroimaging has been used to monitor the clinical progress of these patients, especially in deteriorating cases. In order to decrease morbidity and mortality, an understanding of CVST treatment options is important. Treatment of extensive intracranial venous sinus thrombosis with intrasinus infusion of recombinant tissue plasminogen activator (rt-PA) is relatively controversial as there are no clear guidelines in regards to appropriate therapeutic management. We report a case of successful intrasinus thrombolysis of deep cerebral sinus thrombosis (DCST) resulting in rapid radiographic improvement associated with complete clinical recovery.

## 1. Case Description

**Figure 1:**
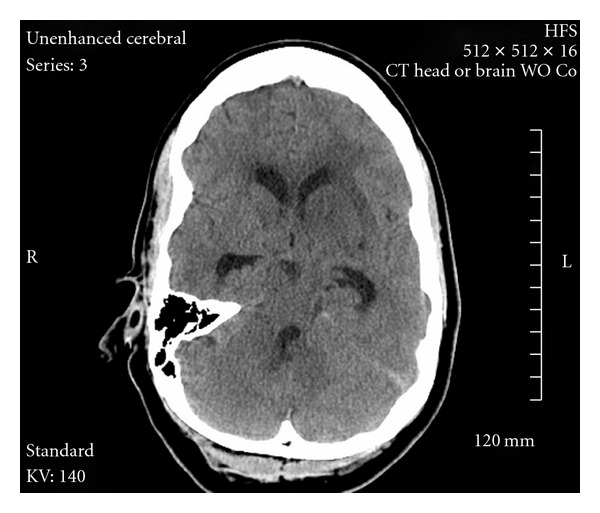
Noncontrast CT scan after 24 hours of intravenous Heparin.

A 43-year-old right-handed woman with past medical history of diabetes, hypertension, and concurrent use of oral contraceptives presented to hospital with sudden onset of nausea, vomiting, and headache, followed by a decreased level of consciousness. Initial noncontrast brain CT showed hyperdense areas along the straight and sagittal sinuses, suggestive of DCST, later confirmed by magnetic resonance venography (MRV) (Figure 4). Hence, intravenous heparin 1000 units/hour was started with goal PTT of 79 achieved by noon the following day. However, after 24 hours of continuous heparin infusion, she began to show signs of neurological deterioration with right hemiparesis, dysarthria, global aphasia, and depressed levels of consciousness. A second CT of the brain was obtained which revealed persistent hyperdensities in the straight sinus and deep cerebral veins, with new hypodense regions involving bilateral thalami and basal ganglia likely representing edema and venous infarction (Figure 1). Patient was immediately transferred to the cerebral angiography suite for intrasinus thrombolysis (IST) with rt-PA.

**Figure 2:**
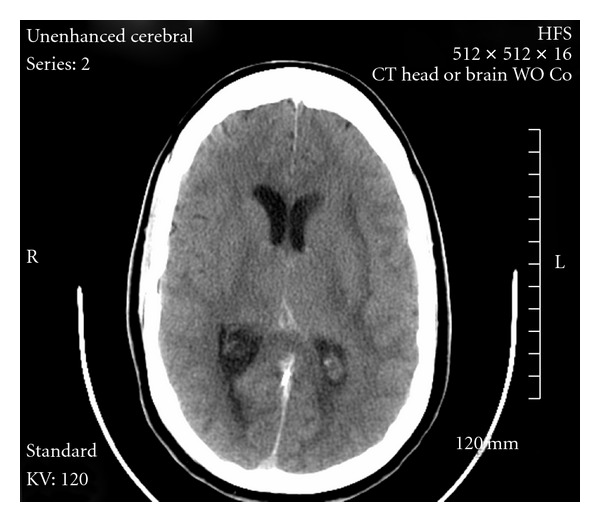
Noncontrast CT scan 1 hour after intrasinus rt-PA.

**Figure 3:**
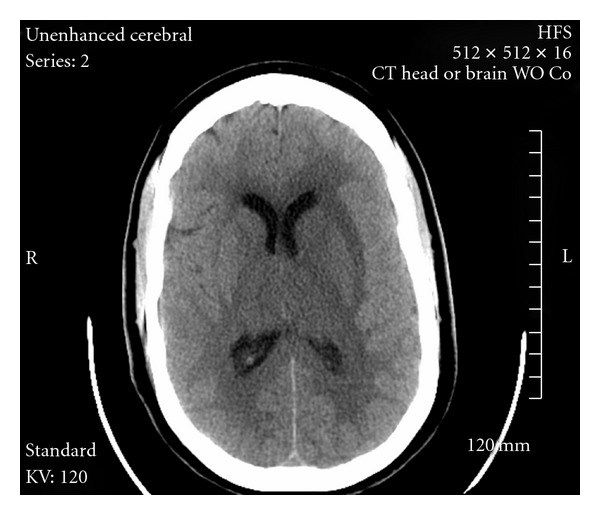
Noncontrast CT scan after 24 hours of intrasinus rt-PA.

**Figure 4:**
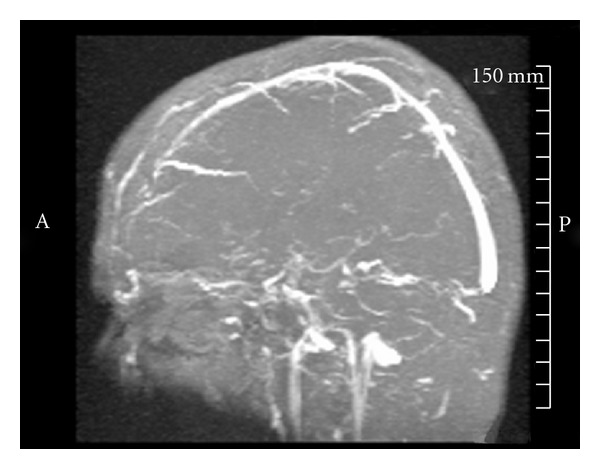
MRV on admission.

Left transverse sinus and straight sinus were catheterized via transfemoral route and then retrograde catheterization was done via the left jugular vein with the tip of the microcatheter advanced to the superior aspect of the left straight sinus. A bolus of 10 mg rt-PA was given, followed by overnight infusion of rt-PA at 1 mg/hr. Five hours after the initial angiogram, repeat CT had shown improvement in the thalamic and basal ganglia edema (Figure 2). After fourteen hours of infusion, the patient had a follow-up angiogram the next morning, which showed the straight sinus to be widely patent. The microcatheter was then readjusted towards the medial aspect of left transverse sinus, and intrasinus rt-PA infusion was continued. A repeat angiogram revealed substantially improved flow within the deep/subependymal venous system, left transverse sinus and straight sinus. Thrombolytic infusion was stopped after twenty-two hours of total infusion with heparin restarted, followed by warfarin. Another follow-up CT scan was done twelve hours later, which did not show any evidence of cortical infarction or hemorrhage (Figure 3).

**Figure 5:**
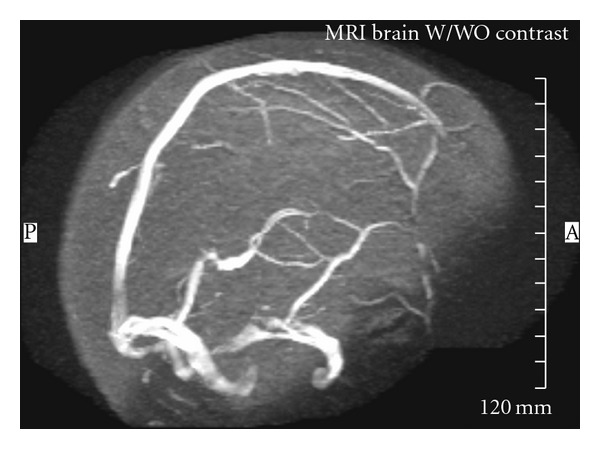
MRV at 1 month follow-up after rt-PA.

On day four, the patient had no apparent neurological deficits, followed commands and moved all extremities equally and was extubated. She improved over the next nine days of her hospital stay and on discharge her NIHSS score was 2 (slight receptive aphasia and limb ataxia). The search for a hypercoagulable etiology was unremarkable. After one month follow-up MRI and MRV revealed complete patency of the venous system without residual infarct (Figure 5). Neurological exam showed no deficits and an NIHSS score of 0 and a modified Rankin score of 0. 

## 2. Discussion

Deep cerebral venous thrombosis is associated with a grave prognosis, with many cases diagnosed postmortem. Clinical diagnosis is often quite confusing due to the great variability in the clinical presentation of the disease and low clinical suspicion. This emphasizes the importance of radiological diagnosis [1, 2]. The natural history of patients with DCSTs is highly variable with mortality rates of 30–50% (36% in DCST and 11% in superficial thrombosis) [3]. With clinical presentation varying from headache in 95%, focal seizures in 47%, unilateral or bilateral paresis in 43%, and papilledema in 43%, the initial diagnosis and beginning of early treatment difficult [3]. Not uncommonly, patient may deteriorate to a comatose state despite anticoagulation therapy, as in our patient.

Scott et al. in 1988 reported the first use of local thrombolysis with infusion of urokinase [4]. This was followed by many case series using rt-PA. Two uncontrolled studies, totaling 21 subjects, used rt-PA in combination with dose-adjusted intravenous heparin [5, 6]. In both studies, a microcatheter was placed directly into the thrombus via a transfemoral venous approach and rt-PA bolus followed by continuous infusion ensued. Fourteen out of twenty-one subjects (67%) showed complete recovery [5, 6]. Adverse events cited in both studies included intracerebral hemorrhage (ICH) but was similar to that seen with systemic intravenous rt-PA for ischemic cerebral infarction. 

Ciccone et al. performed a systematic review of intrasinus thrombolysis (IST) for CVST [7]. Patients were either treated with heparin and urokinase or heparin and rt-PA, which may carry less bleeding complications due to its clot selectiveness and shorter half-life [7, 8]. Their conclusion was that as there are currently no randomized controlled trials (RCTs) regarding the efficacy or safety of thrombolytic therapy in CVST; this is not the standard of treatment even in deteriorating cases. We are not aware of any specific reports documenting such an early, dramatic improvement in cerebral imaging. In outpatient, the improvement in cerebral imaging occurred many hours prior to the first clinical signs of improvement. In another study, 19 patients with CVST received IST. At discharge, 15 patients (79%) had good outcome and were either asymptomatic or had only mild deficits and were independent for activities of daily living [9].

Despite the lack of RCTs, there are many uncontrolled studies with the use of combined heparin and selective intrasinus rt-PA in CVST, all of which have been very encouraging despite the risk of ICH. This treatment appears effective and safe, given the overall poor prognosis, especially with deep cerebral venous involvement. The excellent clinical response associated with rapid radiological improvement, as in our patient, makes it especially appealing as a treatment modality.
